# Effects of Dietary Rumen-Protected Betaine Supplementation on Performance of Postpartum Dairy Cows and Immunity of Newborn Calves

**DOI:** 10.3390/ani9040167

**Published:** 2019-04-15

**Authors:** Beibei Wang, Chong Wang, Ruowei Guan, Kai Shi, Zihai Wei, Jianxin Liu, Hongyun Liu

**Affiliations:** 1 College of Animal Sciences, Zhejiang University, Hangzhou 310058, China; 21617083@zju.edu.cn (B.W.); 21617025@zju.edu.cn (R.G.); shik_1992@163.com (K.S.); weizihai365@163.com (Z.W.); liujx@zju.edu.cn (J.L.); 2 College of Animal Sciences and Technology, Zhejiang A & F University, Hangzhou 311300, China; wangcong992@163.com

**Keywords:** betaine, dairy cows, newborn calves, fat mobilization, immunity

## Abstract

**Simple Summary:**

Betaine plays an important role in growth, lactation, protein synthesis, and fat metabolism in animals, but there are few studies on transition dairy cows and newborn calves. The aim of the current study was to evaluate the effects of rumen-protected betaine supplementation from four weeks before expected calving to six weeks postpartum regarding the lactation performance and blood metabolites of dairy cows and immunity of newborn calves. The results suggested that betaine supplementation tended to increase fat mobilization of postpartum dairy cows. Furthermore, compared to the control calves, the betaine calves had greater plasma total protein and globulin concentrations, which indicates that the immunity of the betaine calves might have improved.

**Abstract:**

The objective of this study was to evaluate the effects of rumen-protected betaine supplementation on performance of postpartum dairy cows and immunity of newborn calves. Twenty-four multiparous Holstein dairy cows were randomly divided into the control (CON, *n* = 12) and rumen-protected betaine (BET, *n* = 12) groups after blocking by parity and milk yield during the previous lactation cycle. The cows were fed a basal total mixed ration diet without BET (CON) or with BET at 20 g/d per cow (BET) from four weeks before expected calving to six weeks postpartum. The results showed that betaine supplementation had no effect on dry matter intake and milk yield of the cows. The BET cows tended to increase feed efficiency (energy-corrected milk/dry matter intake) and body weight loss postpartum compared to the CON cows. The plasma β-hydroxybutyrate concentrations of the BET cows were greater at d seven after calving than those of the CON cows. Moreover, compared to the CON calves, the BET calves had greater plasma total protein and globulin concentrations. The plasma glucose concentrations of the BET calves tended to decrease relative to CON cows. In conclusion, rumen-protected betaine supplementation from four weeks before expected calving tended to increase fat mobilization of postpartum dairy cows, and might improve the immunity of newborn calves.

## 1. Introduction

During the transition period, dairy cows are in a state of great metabolic stress because of the increased demand for nutrients to maintain fetal growth and milk synthesis. Transition dairy cows tend to have negative energy and amino acid balance after calving, which leads to an increase in fat and protein mobilization in tissues [1,2]. A negative methyl donor balance also likely occurs in transition cows because milk is high in methylated compounds [3]. Moreover, the last two months of gestation, where 60% of the body weight gain before birth occurs [4], is critical for bovine fetal development.

Betaine functions as a methyl donor and an organic osmolyte [2,5], which plays an important role in growth, lactation, protein synthesis, and fat metabolism in animals [6]. Betaine supplementation in the diets of steers increased body weight gain and fat deposition [7]. In lactating dairy cows, feeding betaine improved the milk yield and milk protein [8,9]. Supplementing betaine reduced plasma concentrations of non-esterified fatty acids (NEFA) and β-hydroxybutyrate (BHB) of lactating dairy cows [10], but elevated the concentrations of NEFA and BHB of transition dairy cows to change lipid metabolism [11]. Furthermore, betaine is vital for fetal development [12], and is related to the offspring’s weight and immunity [13]. However, due to the fast rumen degradation (approximately 45%/h) of betaine in vivo [14], unprotected betaine cannot be absorbed efficiently. Our previous study showed that dietary rumen-protected betaine supplementation in lactating dairy cows improved lactation performance and fat metabolism [15]. Whether it improves the performance of postpartum dairy cows and the immunity of newborn calves remains unexplored. Therefore, the objectives of the current study were to evaluate the effects of rumen-protected betaine supplementation from four weeks before expected calving to six weeks postpartum on the lactation performance and blood metabolites of dairy cows and immunity of newborn calves.

## 2. Materials and Methods 

### 2.1. Animals and Treatments

All the experimental protocols used in this study were approved by the Animal Care Committee of Zhejiang University (Hangzhou, China) (Approval Number: ZJU20160379). Twenty-four multiparous prepartum Holstein dairy cows were selected and divided randomly into the control (CON, *n* = 12) and rumen-protected betaine (BET, *n* = 12) groups after blocking by parity (2.27, SD = 1.4) and milk yield during the previous lactation cycle (24.9 kg/d, SD = 6.0). The cows were fed a basal total mixed ration (TMR) diet (Table 1) without BET (CON) or with BET at 20 g/d per cow (BET), according to Zhang et al. (2014) [9] from four weeks before expected calving to six weeks postpartum. The basal diets were formulated based on the NRC (2001) [16]. The cows were fed three times daily at approximately 06:30, 13:30, and 19:30 h, and BET (BET with 30% purity, Hangzhou King Technology Feed Co., Ltd, Hangzhou, China) was supplemented twice daily in the morning and evening by top-dressing the TMR during feeding. All cows were housed in tie-stall barns and given access to fresh water ad libitum. After parturition, the cows were milked three times daily at approximately 07:00, 14:00, and 20:00 h. Fourteen calves randomly selected (CON calves: *n* = 7, BET calves: *n* = 7) were studied from birth to 24 h. All calves were weighed with a digital scale immediately after birth and were fed fresh colostrum from their dams within 2 h of birth. The calves were individually housed in hutches, and water was offered ad libitum. 

### 2.2. Sample Preparation

The amounts of feed offered and refused were recorded according to Gu et al. (2018) [17] to determine dry matter intake (DMI). The TMR samples were collected weekly for dry matter (DM, 105 °C for 5 h), crude ash, ether extract (EE), crude protein (CP), and acid detergent fiber (ADF), according to AOAC procedures (method 942.05, 920.39, 988.05, and 973.18, respectively), and neutral detergent fiber (NDF) with sodium sulfite and amylase was analyzed [18]. Body weight (BW) was measured on d 0 and 42 after calving according to Wang et al. (2017) [19]. 

Milk yield was recorded for two consecutive days each week and milk samples from three consecutive milking were taken each week in the amounts proportional to the yield (4:3:3, composite from each daily milking). The samples were stored at 4 °C with bronopol tablets (D & F Control System Inc., San Ramon CA, USA) for later determination of protein, fat, lactose, total solids, and milk urea nitrogen (MUN) using a Combi Foss FT+ instrument (Foss Electric, Hillerød, Denmark). The 3.5% FCM (Fat-corrected milk) and ECM (Energy-corrected milk) were calculated by the formula [20]: 3.5% FCM = (milk yield, kg/d × 0.4324) + (milk fat, kg/d × 16.216), ECM = (milk yield, kg/d × 0.327) + (milk fat, kg/d × 12.95) + (milk protein, kg/d × 7.20).

Blood samples from the cows were collected from the coccygeal vein in sodium-heparinized tubes at approximately 4 h after the morning feeding on −21, −10, 0, 7, 14, 28, and 42 d relative to calving. Blood samples from the calves were collected via the jugular vein using a sodium-heparinized tube shortly after birth before colostrum feeding and at approximately 24 h after birth. The samples were centrifuged for 10 min at 3000 g at 4 °C to harvest plasma, which was stored at -20 °C until analysis. The plasma samples were analyzed using an Auto Analyzer 7020 instrument (Hitachi High-Technologies Corp., Tokyo, Japan) with colorimetric commercial kits (Ningbo Medical System Biotechnology Co., Ltd., Ningbo, China) for total protein (TP), albumin (ALB), alanine aminotransferase (ALT), aspartate aminotransferase (AST), alkaline phosphatase (ALP), total bilirubin (TBIL), triglyceride (TG), cholesterol (CHOL), glucose (GLU), superoxide dismutase (SOD), NEFA, and BHB. The concentrations of globulin (GLOB) were calculated by the formula [21]: GLOB (g/L) = TP (g/L) − ALB (g/L).

### 2.3. Data Analysis

A randomized block design with repeated measures was used. The DMI, lactation performance, feed efficiency, and blood metabolites of the cows were analyzed with PROC MIXED of SAS 9.2 (SAS Institute Inc., Cary, NC, USA). Treatment, week, treatment × week, and block were included as the fixed effects in the model, and cow within treatment was used as a random effect. The blood metabolites of the calves were analyzed using the same procedure in SAS 9.2, except sampling hour instead of week was used as the repeated measure. The BW change of the cows, colostrum composition, and calves birth weight were analyzed using PROC MIXED of SAS 9.2 without the repeated statement. All associated interactions were removed from the model. The results are presented as least squares means. Statistical significance was determined at *p* ≤ 0.05 and tendencies at 0.05 < *p* ≤ 0.10.

## 3. Results and Discussion

Betaine supplementation had no effect on DMI, milk yield, and composition (*p* > 0.1, Table 2). Monteiro et al. (2017) [11] found that cows supplemented with betaine-containing molasses from 60 d before expected calving had higher milk yield, whereas no differences were observed in milk yield of cows supplemented with betaine-containing molasses from 24 d before expected calving, which is consistent with our results. The addition of betaine during the transition period increased the milk yield in a time-dependent manner, which might be related to the functions of betaine as an organic osmolyte to maintain the cell function by stabilizing cellular proteins and promoting proper protein folding [5,22]. The dry period is critical for the renewal and growth of mammary cells [23]. Hence, betaine addition during the far-off period has a positive effect on prepartum mammary growth, which increases the subsequent milk yield. The Lys: Met ratio in the postpartum diets was estimated to be 3.13:1 in our study, which had met the ideal Lys: Met ratio of 3.0:1 for an optimal milk protein content and yield [16,24]. Methyl donors (choline) additional supplementation had no detectable effect on cow performance when the Lys: Met ratio in diets had reached 3.0:1 [25]. This might also be a reason for BET additional supplementation, which has no effect on milk yield and composition. 

Compared to CON cows, BET cows tended to increase fat-corrected milk/dry matter intake (FCM/DMI, *p* = 0.09), energy-corrected milk/dry matter intake (ECM/DMI, *p* = 0.08), and BW loss postpartum (*p* = 0.10) (Table 2). The plasma BHB concentrations of the BET cows were greater at d seven after calving than those of the CON cows (treatment × time: *p* = 0.07, Table 3). The greater number of animals in the study might have increased the statistical significance. The BET cows tended to have greater feed efficiency and BW loss postpartum in our study, coupled with greater concentrations of BHB at d seven after calving, which indicates that the BET cows might have an enhanced fat mobilization in early lactation due to higher milk yield numerically (milk yield was approximately 2.53 kg/d higher in BET cows than in CON cows) [11,26].

The plasma TP and GLOB concentrations of the BET calves were greater than those of the CON calves (*p* = 0.04, *p* = 0.05, respectively, Table 4), although no differences in calves birth weight were found between treatments (37.80 ± 1.68 kg vs. 36.03 ± 1.68 kg). The plasma TP and GLOB concentrations of calves increased significantly with maternal betaine supplementation, which indicates that it might improve the immunity of newborn calves because of maternal methyl donors supplementation [13,27]. Maternal dietary supplementation with methyl donors could program the health of offspring through the epigenetic regulation of the DNA molecule and cell signaling [27,28], which might improve the capacity for GLOB absorption of the intestine to improve the immunity of newborn calves. Furthermore, the lactose content of the BET colostrum tended to increase compared to the CON colostrum in our study (3.50% vs. 3.07%, *p* = 0.07), which might also have contributed to the results. Lactose plays a key role in the energy supply, absorption of minerals, and gastrointestinal functions of calves [29,30]. 

The plasma GLU concentrations of the BET calves tended to decrease compared with those of the CON calves (*p* = 0.09, Table 4). The plasma SOD concentrations of the BET calves were greater at 2 h after birth than those of the CON cows (treatment × time: *p* = 0.01, Table 4). A positive correlation between neonatal glucose and cortisol concentrations proved that the lower concentrations of glucose in the BET calves were likely to have less stress around calving [31]. In turn, the degree of stress might influence newborn calves’ energetic mobilization [31,32]. The greater plasma SOD concentrations 2 h after birth in our study suggested that the BET calves were in a state of less stress [33]. The plasma glucose concentrations in newborn calves might be related to the uteroplacental transport of glucose via mTOR signaling [34] and hepatic gluconeogenic gene expression via epigenetic mechanisms [35], which deserve further study.

## 4. Conclusions

Dietary rumen-protected betaine supplementation from four weeks before expected calving had no detectable effect on dry matter intake and milk yield, but tended to increase fat mobilization of postpartum dairy cows. Furthermore, the BET calves had greater plasma total protein and globulin concentrations, which indicates that the immunity of the BET calves might improve.

## Figures and Tables

**Table 1:** Ingredient and chemical composition of the diets fed during the prepartum and postpartum periods.

Item	Prepartum	Postpartum
Ingredient, % of DM ^1^		
Corn flour	12.41	13.39
Steam-flaked corn	7.15	11.93
Soybean meal	8.61	13.68
Bran	5.30	-
Sodium bicarbonate	0.40	0.72
Calcium hydrophosphate	0.40	0.48
Limestone	0.60	0.66
Fatty acid calcium salts	-	0.78
Salt	0.39	0.46
Premix ^2^	0.37	0.44
Mycotoxin binder	0.05	0.07
Active yeast	-	0.07
Brewer’s grains	7.31	4.55
Beet pulp	6.70	9.26
Corn silage	25.39	21.05
Alfalfa hay	6.78	16.86
Oat grass	18.08	5.62
Chemical composition, % of DM		
Crude protein	10.99	17.49
Ether extract	3.27	4.31
Crude ash	7.96	7.84
Neutral detergent fiber	48.48	37.08
Acid detergent fiber	27.57	20.78
NEL, Mcal/kg of DM	-	1.63
Lys: Met	2.76:1	3.13:1

^1^ DM = dry matter.^2^ Formulated to contain (per kilogram of premix) 220 to 400 KIU of vitamin A, 50 to 100 KIU of vitamin D3, ≥2250 IU of vitamin E, ≥40 mg of D-Biotin, ≥380 mg of niacinamide, ≥40 mg of Beta-carotene, 0.2 to 0.7 g of Cu, 1.0 to 3.8 g of Zn, 0.8 to 3.0 g of Mn, 12.5 to 100 mg of I, 8.0 to 25 mg of Se, 2.5 to 50 of mg Co, 10.0% to 30.0% of Ca, 10.0% to 30.0% of NaCl, and ≥1.5% of total phosphorus.

**Table 2:** Effects of supplementing cows without rumen-protected betaine (CON) or with rumen-protected betaine (BET) on dry matter intake, lactation performance, and body weight change during the first six weeks of lactation.

Items	Treatment		SEM	*p*-Value		
	CON	BET		Treat	Week	Treat × Week
DMI, kg/d	20.33	20.21	0.76	0.92	<0.01	0.10
Milk yield, kg/d	30.44	32.97	1.68	0.31	<0.01	0.61
Milk composition						
Fat, %	4.30	4.18	0.09	0.35	<0.01	0.44
Protein, %	3.23	3.13	0.06	0.30	<0.01	0.71
Lactose, %	5.00	4.94	0.04	0.28	0.00	0.50
Total solids, %	12.93	12.77	0.13	0.42	<0.01	0.93
MUN, mgN/dL	10.74	10.64	0.63	0.91	0.00	0.23
3.5% FCM ^1^, kg/d	34.36	36.35	1.83	0.46	0.05	0.49
ECM ^2^, kg/d	34.24	35.70	1.79	0.58	0.33	0.38
FE (FCM/DMI)	1.80	2.00	0.08	0.09	<0.01	0.22
FE (ECM/DMI)	1.77	1.96	0.07	0.08	<0.01	0.26
BW change, kg/d	−1.18	−1.51	0.13	0.10	-	-

^1^ 3.5% FCM (Fat-corrected milk) = (milk yield, kg/d × 0.4324) + (milk fat, kg/d × 16.216) [20]. ^2^ ECM = (milk yield, kg/d × 0.327) + (milk fat, kg/d × 12.95) + (milk protein, kg/d × 7.20) [20].

**Table 3:** Effects of supplementing cows without rumen-protected betaine (CON) or with rumen-protected betaine (BET) on blood metabolites from four weeks before expected calving to six weeks postpartum.

Items ^1^	Treatment		SEM	*p*-Value		
	CON	BET		Treat	Week	Treat × Week
TP, g/L	78.78	78.95	1.61	0.94	<0.01	0.36
ALB, g/L	25.77	25.42	0.35	0.50	<0.01	0.28
GLOB, g/L	53.01	53.53	2.02	0.86	<0.01	0.56
A/G	0.50	0.49	0.02	0.76	<0.01	0.76
ALT, U/L	14.41	13.90	0.94	0.71	<0.01	0.81
AST, U/L	71.86	73.67	4.15	0.76	<0.01	0.40
ALP, U/L	33.29	36.34	1.81	0.26	<0.01	0.91
TBIL, µmol/L	2.61	2.75	0.25	0.70	<0.01	0.99
TG, mmol/L	0.08	0.08	0.01	0.32	<0.01	0.39
CHOl, mmol/L	2.52	2.37	0.09	0.26	<0.01	0.42
GLU, mmol/L	3.34	3.30	0.07	0.72	<0.01	0.83
NEFA, µmol/L	246.57	243.90	20.74	0.93	<0.01	0.95
BHB, µmol/L	802.72	812.65	92.55	0.94	<0.01	0.07

^1^ TP = total protein. ALB = albumin. GLOB = globulin. A/G = albumin/globulin. ALT = alanine aminotransferase. AST = aspartate aminotransferase. ALP = alkaline phosphatase. TBIL = total bilirubin. TG = triglyceride. CHOL = cholesterol. GLU = glucose. NEFA = non-esterified fatty acids. BHB = β-hydroxybutyrate.

**Table 4:** The blood metabolites during the 24 h after birth of calves born to dams supplemented without rumen-protected betaine (CON) or with rumen-protected betaine (BET) during the peripartal period.

Items ^1^	Treatment		SEM	*p*-Value		
	CON Calves	BET Calves		Treat	Hour	Treat × Hour
TP, g/L	54.71	59.63	1.48	0.04	<0.01	0.15
ALB, g/L	18.48	18.59	0.42	0.85	0.00	0.03
GLOB, g/L	36.23	41.03	1.57	0.05	<0.01	0.06
A/G	0.58	0.56	0.02	0.54	<0.01	0.04
ALT, U/L	7.67	8.24	0.37	0.30	<0.01	0.21
AST, U/L	53.67	55.31	4.98	0.82	<0.01	0.87
ALP, U/L	265.38	257.78	35.73	0.88	<0.01	0.86
TBIL, µmol/L	6.21	6.60	0.87	0.76	0.05	0.97
GLU, mmol/L	5.62	4.82	0.30	0.09	0.00	0.36
SOD, U/ml	66.54	70.52	2.91	0.35	0.00	0.01

^1^ TP = total protein. ALB = albumin. GLOB = globulin. A/G = albumin/globulin. ALT = alanine aminotransferase. AST = aspartate aminotransferase. ALP = alkaline phosphatase. TBIL = total bilirubin. GLU = glucose. SOD = superoxide dismutase.
